# Epigenetic silencing of *AATK* in acinar to ductal metaplasia in murine model of pancreatic cancer

**DOI:** 10.1186/s13148-020-00878-6

**Published:** 2020-06-17

**Authors:** Li-Yun Ding, Ya-Chin Hou, I-Ying Kuo, Ting-Yi Hsu, Tsung-Ching Tsai, Hsiu-Wei Chang, Wei-Yu Hsu, Chih-Chieh Tsao, Chung-Chen Tian, Po-Shun Wang, Hao-Chen Wang, Chung-Ta Lee, Yi-Ching Wang, Sheng-Hsiang Lin, Michael W. Hughes, Woei-Jer Chuang, Pei-Jung Lu, Yan-Shen Shan, Po-Hsien Huang

**Affiliations:** 1 Department of Biochemistry and Molecular Biology, College of Medicine, National Cheng Kung University, Tainan, Taiwan; 2 Institute of Basic Medical Sciences, College of Medicine, National Cheng Kung University, Tainan, Taiwan; 3 Institute of Clinical Medicine, College of Medicine, National Cheng Kung University, Tainan, Taiwan; 4 Department of Pharmacology, College of Medicine, National Cheng Kung University, Tainan, Taiwan; 5 Department of Pathology, National Cheng Kung University Hospital, College of Medicine, National Cheng Kung University, Tainan, Taiwan; 6 Department of Public Health, College of Medicine, National Cheng Kung University, Tainan, Taiwan; 7 Biostatistics Consulting Center, National Cheng Kung University Hospital, College of Medicine, National Cheng Kung University, Tainan, Taiwan; 8 International Center for Wound Repair & Regeneration, National Cheng Kung University, Tainan, Taiwan; 9 Department of Surgery, National Cheng Kung University Hospital, College of Medicine, National Cheng Kung University, Tainan, Taiwan

**Keywords:** AATK, TP63, DNA methylation, KPC model, Pancreatic cancer

## Abstract

**Background:**

Cancer subtype switching, which involves unclear cancer cell origin, cell fate decision, and transdifferentiation of cells within a confined tumor microenvironment, remains a major problem in pancreatic cancer (PDA).

**Results:**

By analyzing PDA subtypes in The Cancer Genome Atlas, we identified that epigenetic silencing of apoptosis-associated tyrosine kinase (*AATK*) inversely was correlated with mRNA expression and was enriched in the quasi-mesenchymal cancer subtype. By comparing early mouse pancreatic lesions, the non-invasive regions showed AATK co-expression in cells with acinar-to-ductal metaplasia, nuclear VAV1 localization, and cell cycle suppression; but the invasive lesions conversely revealed diminished AATK expression in those with poorly differentiated histology, cytosolic VAV1 localization, and co-expression of p63 and HNF1α. Transiently activated AATK initiates acinar differentiation into a ductal cell fate to establish apical-basal polarization in acinar-to-ductal metaplasia. Silenced AATK and ectopically expressed p63 and HNF1α allow the proliferation of ductal PanINs in mice.

**Conclusion:**

Epigenetic silencing of *AATK* regulates the cellular transdifferentiation, proliferation, and cell cycle progression in converting PDA-subtypes.

## Introduction

Understanding the biological behaviors and molecular alterations that occur during the progression from pancreatic intraepithelial neoplasia (PanIN) to pancreatic ductal adenocarcinoma (PDA) is essential for the identification of clinically relevant biomarkers for early detection and diagnosis, the development of preventive and therapeutic strategies, and the control of PDA progression [1]. Collisson et al. [2] evaluated the gene expression profiles of microdissected PDA samples and categorized PDA initially into three subtypes, “classical” [3], “quasi-mesenchymal” (QM-PDA), and exocrine-like [4], which all correlate with clinical outcome [2, 5, 6] (reviewed in [7]). An increasing number of studies then compared the differences in the stroma, immune cell infiltration, and metabolic alterations to further elaborate on the classical, the basal-like [8–11], and the squamous cells that closely resemble the QM-PDA subtype [10, 12–15], which is associated with the worst survival outcome. Exocrine-like PDAs are HNF1α^+^, as determined by immunostaining, and are frequently resistant to paclitaxel and tyrosine kinase inhibitors due to accelerated drug metabolization [10]. HNF1α directs the transcription of oncogenic cancer stemness genes [16] and correlates with a reduced survival in patients. Aberrant expression of transcription factors, including PDX1, PTF1A, and HNF1A, has been linked to PDA and subtype progression [5, 17]. TRP63 functionally reprograms oncogenic enhancers in squamous PDA [12, 13]. HNF1A interacts with the *NR5A2* promoter and in turn regulates acinar gene expression, acinar cell differentiation, and acinar homeostasis [18]. SOX9 is involved in the programming of pancreatic progenitors [19] and is present in terminal-differentiated ductal cells [20]. Although the epigenetic landscape of PDA subtypes has been described [14], the developmental roles of subtype-specific suppressor gene signatures gene expression patterns in tissue development and homeostasis have not been thoroughly studied.

Recurrent mutations in the *KRAS* oncogene and in a number of tumor suppressor genes, including *TP53*, *CDKN2A*, and *SMAD4*, have been identified in pancreatic cancer [21]. Genomic analyses have revealed that an activating *KRAS* mutations are present early in the pancreatic PanIN precursor lesions in the ductal epithelium of the pancreas. Key mouse models, including the *Kras*^*G12D/+*^; *Trp53*^*fl/+*^; *Pdx1*-*Cre* (KPC) model, have been established for understanding the initiation, development, progression, and metastasis of PDA [22–25]. Collectively, they encompass genome instability [26], clonal expansion [27], hereditary [28], or environmental pathways. PanINs may develop into cancer through multistep tumorigenesis, or it has been hypothesized to transdifferentiate into invasive cancer cells that have mesenchymal properties directly within the primary tumors. Aberrantly expressed cancer genes are frequently marked by aberrant DNA methylation, and this process signifies the dysregulation of the epigenetic states in committed adult somatic tissue cells. As promoter hypermethylation of tumor suppressor genes provides transcriptional silencing, hypo-methylation of proto-oncogenes through transcriptional activation has been shown to play important roles in cancers [29]. Pancreatic cancer remains a highly lethal malignancy, with a 5-year survival rate of less than 8%, and pancreatic ductal adenocarcinomas (PDA) account for more than 95% of all pancreatic tumors [30]. In total, 74% of the patients succumb to this disease within a year after diagnosis and have a median survival of less than 6 months [31]. Patients with localized disease exhibit no overt symptoms, and few screening approaches can accurately detect PDA at early stages.

The apoptosis-associated tyrosine kinase (*AATK*) gene is located at 17q25.3 [32], a frequent LOH region in the chromosome 17q arm of pancreatic cancer samples characterized by array CGH and sequencing [33–35]. This locus encodes two AATK tyrosine kinase isoforms [36] and one *AATK* antisense transcript (AATK-AS). AATK promotes neuronal differentiation [37], axon outgrowth [38], and interacts with the cytoskeleton [39] in neuronal cells. In melanoma cell lines, AATK suppresses growth and migration and promotes apoptosis [40]. However, the expression of *AATK* and its functional role in the course of PDA initiation, progression, and clinical outcome have not been determined in large clinical cohorts, despite its potential apoptosis-promoting role in other cancers [40, 41]. Therefore, understanding its biological behavior and molecular function during progression prior to PDA development will help characterize the associated molecular subtype of pancreatic cancer development. In the present study, high-throughput promoter methylation analysis of the *AATK* gene was able to distinguish epigenetically silenced *AATK* in our cohort. Protein expression of AATK was inversely correlated with EMT-like cases in our tissue micro-array. siRNA knockdown of AATK expression in pancreatic cancer cell lines led to an upregulation of EMT genes. Overall, our findings provide a novel prognostic marker that can notably discern those patients with the QM subtype from other PDA patients.

## Results

### PDA molecular subtypes associates low expression of AATK with QM-PDA and poor overall survival

**Fig. 1:**
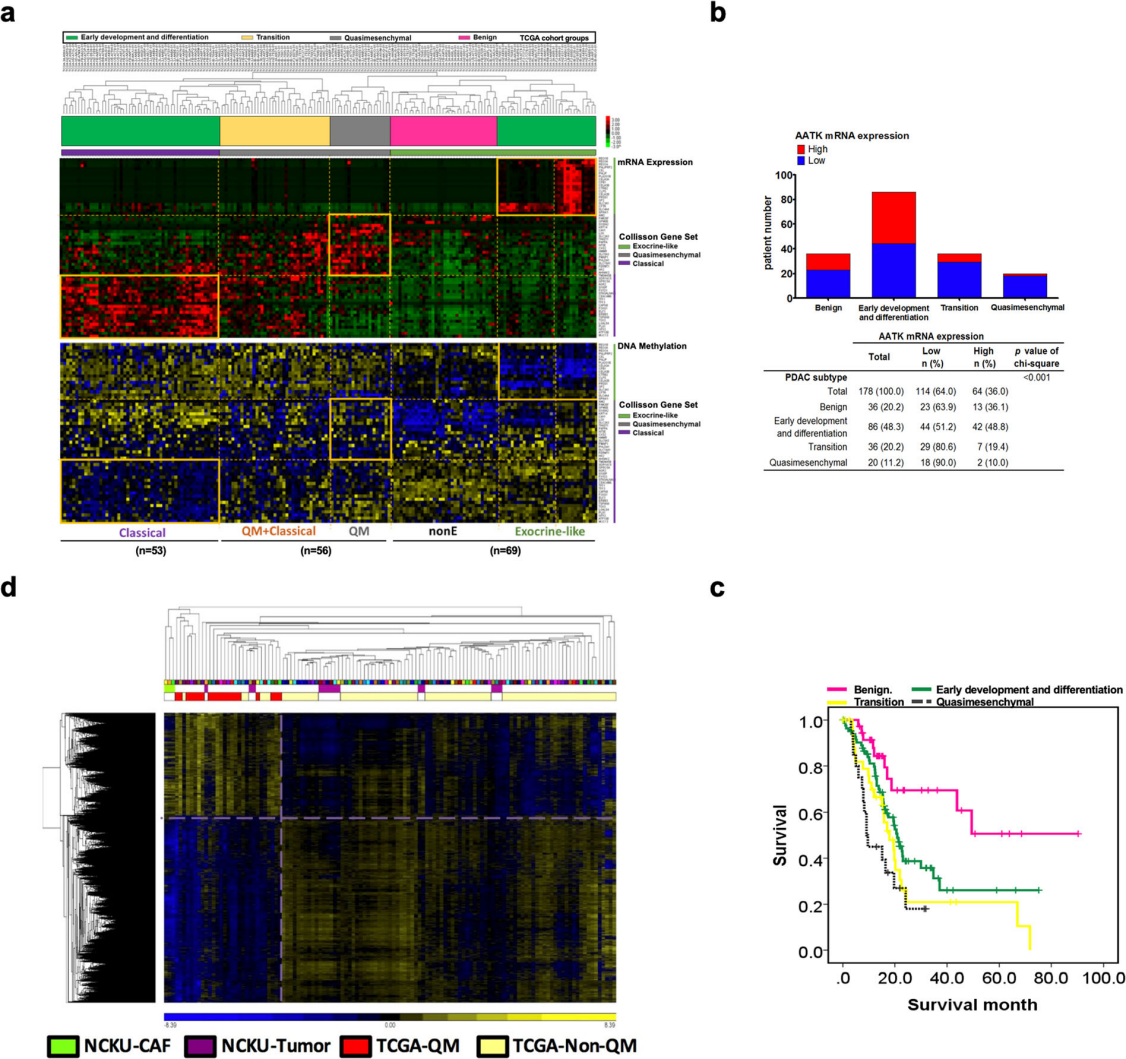
AATK low expression correlated with QM-PDA patients by TCGA database analysis. **a** Heatmap showing three subtypes of PDA in the TCGA dataset. **b** The proportion of patients with low AATK mRNA expression was significantly higher in the QM-PDA patient group. **c** Kaplan–Meier curves indicated that QM-PDA patients had significantly worse overall survival than did non-QM subtype. **d** DNA methylation signature of QM-PDA clusters with primary tumor CAFs. A total of 6134 CpG sites were shown by comparing the QM vs non-QM mRNA expression datasets of TCGA at the level of *p* ≤ 0.05, FDR ≤ 0.01, and ≥ 5% differences in methylation

To compare PDA subtype-specific mRNA expression patterns, we performed an extended analysis to classify these subtypes of PDA patients from the TCGA database. 2D agglomerative clustering of patients consistently identified the three previously characterized PDA subtype classifications in the TCGA database (Fig. 1a), and helped further define the subtype of each specimen in the TCGA dataset. In addition, the Collisson’s subtypes are associated with distinct histopathological characteristics and differential survival rates. Furthermore, the genomic and epigenetic features that characterize each subtype infer different mechanisms of tumorigenesis [2, 12]. In the TCGA data, we used Collisson’s gene signature to classification first (Fig. 1a). The TCGA patients can be found within the same trend but not enough information to distinguish PDA subtypes distinctly. We then separated the patients into four groups, and calculated survival curve for each group: (1) benign (*n* = 36), (2) early development and differentiation (*n* = 86), (3) transition (*n* = 36), and (4) QM-PDA (*n* = 20) based on differential gene expression patterns (Fig. 1a, Fig. S1). With integrated mRNA expression and the DNA methylation data in the TCGA cohort, our analysis identified an inverse correlation between mRNA expression and the DNA methylation patterns in the benign, classical, and exocrine PDAs, but not in the QM-PDA signature genes that are methylated and expressed (Fig. 1a, rectangle boxes). Among QM-PDA individuals, we sought to examine potential subtype-specific suppressors and we then focused on the role and function of *AATK* in PDA subtypes. We analyzed the ratio of AATK expression in each patient group and its association with the QM-PDA subtype. Our analysis showed a significant enrichment of individual patients with underrepresented AATK in the transition subtype (80.6%, 29 out of 36) and in the QM-PDA subtype (90%, 18 out of 20), respectively (chi-square, *p* = 0.001; Fig. 1b). Kaplan–Meier survival estimate analysis of patients from individual classes showed that the QM-PDA subtype indeed had the worst survival outcome of all three subtypes (Fig. 1c). Unsupervised DNA methylation clustering of the QM-PDA cases in TCGA clustered with the methylomes of 100% (three out of three) of patient-derived cancer-associated fibroblasts (CAFs), and 21.4% (3 out of 14) of primary PDA tumors in our cohort. This was based on 6134 CpG sites from the 450K methylation micro-arrays of QM-PDA versus the non-QM-PDA cases dataset, at the level of *p* < 0.05, FDR < 0.01, and 5% difference in DNA methylation β-value (Fig. 1d). These results suggest that AATK low expression might play an important role in the transition mechanism from classical-PDA into QM-PDA.

### Epigenetic silencing of *AATK* associates with downregulated mRNA expression and poor survival subtype of PDA patients

**Fig. 2:**
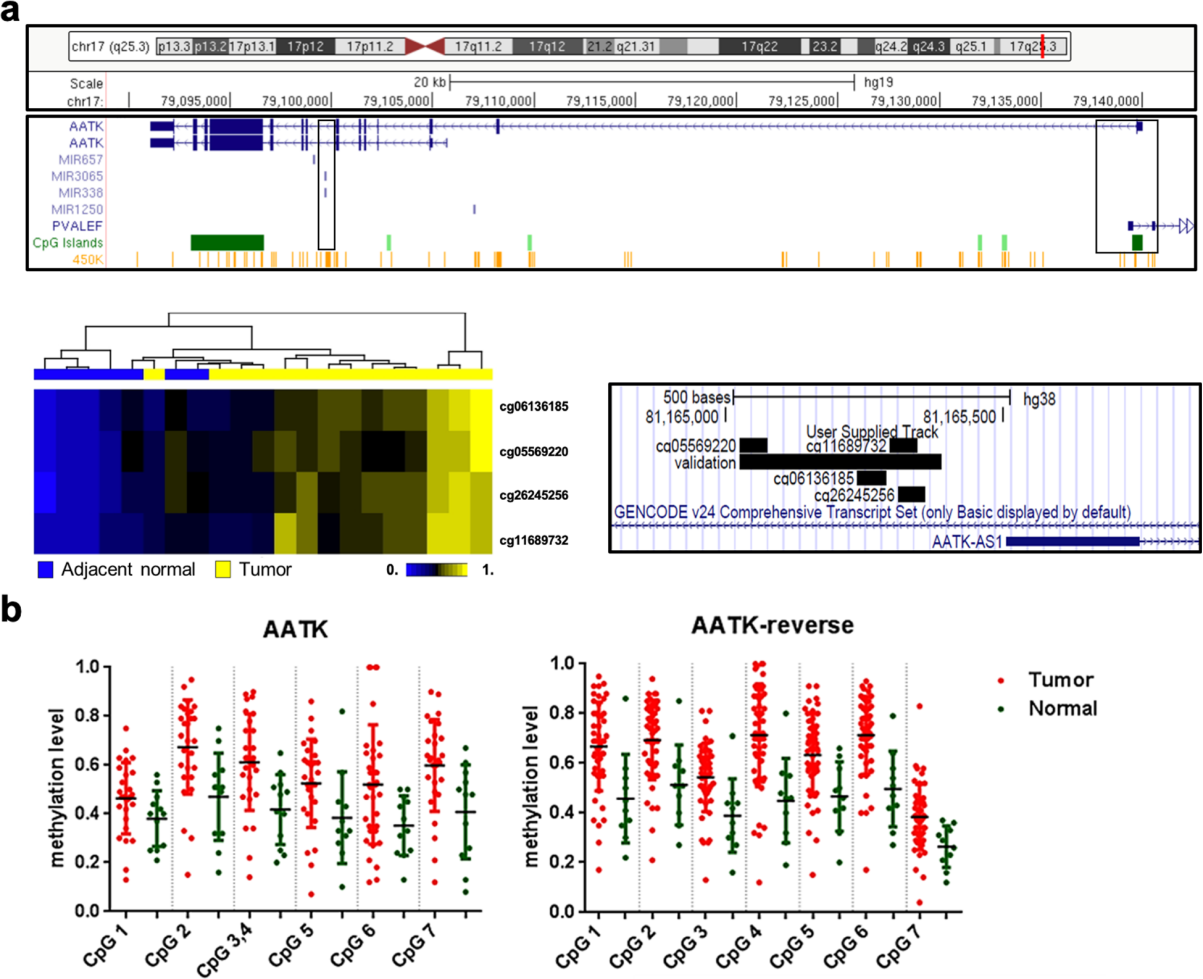
AATK hypermethylation in pancreatic cancer patients. **a** Different methylation probes located in intron 1 of the *AATK* gene as indicated. Heatmap cluster of the four probes in adjacent normal (*N* = 7) and tumor tissue (*N* = 14). The columns indicate individual samples, and the rows indicate different methylated probes. **b** High CpG methylation level of the *AATK* gene in an independent cohort of samples

To identify differentially methylated DNA sites that are associated with cancer progression, we reanalyzed the DNA methylation loci in the HumanMethylation450 micro-arrays from our previous study [42], including 14 tumor samples and seven adjacent normal samples. Based on the β values of the methylation micro-array data, four CpG probes: cg06136185, cg05569220, cg26245256, and cg11689732 located in the CpG island associated with the *AATK* and the *PVALEF* gene promoters, were significantly (*p* < 0.05) hypermethylated in tumors compared to those in adjacent normal tissues (Fig. 2a). To further validate the methylation level of the *AATK* gene in PDA patients from a validation cohort, quantitative DNA methylation analysis spanning seven CpG sites was performed to focus on this differentially methylated region in which these probes were located in the intron 1 of *AATK*. The methylation level of these CpG sites in tumor tissues was significantly increased compared to that in adjacent normal tissues (Fig. 2b). Thus, these data suggest hypermethylation of the first intron of *AATK* in tumors versus normal adjacent tissue, and *AATK* may play an important role in PDA progression.

**Fig. 3:**
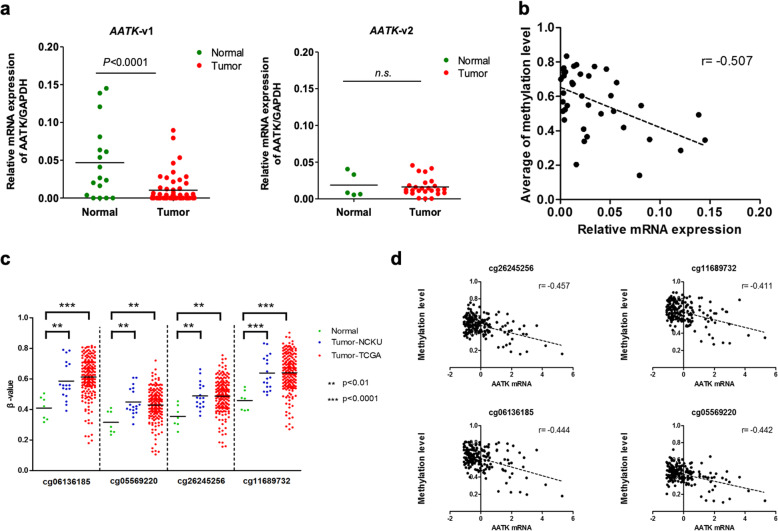
Inverse correlation between DNA methylation and mRNA expression of the *AATK* gene in different cohorts**. a** Dot-plot illustration of the relative mRNA expression levels of *AATK-*variant 1 or variant 2 in normal and tumor tissues of PDA patients. **b** A significant inverse correlation between DNA methylation and mRNA expression was found in PDA patients. **c** DNA methylation level is indicated on the Y-axis and the X-axis represents the adjacent normal and tumor samples from two cohorts with different CpG probes. *P* values were calculated by a two-tailed paired *t* test and are shown as indicated. **d** Inverse correlation between DNA methylation and mRNA expression was found in the TCGA dataset

Next, we aimed to understand the role of promoter hypermethylation in this CpG island on *AATK* gene expression and transcriptional silencing in PDA tumorigenesis. mRNA expression levels of transcripts expressed at this locus were determined by qPCR in PDA patient samples and cell lines, including two AATK transcription variants and one *AATK* antisense transcript (AATK-AS) encoded by the *PVALEF* gene (Fig. 3, S1). The qPCR results indicated that mRNA expression levels of the *AATK-*variant 1 were significantly lower in tumor tissues versus normal tissues from PDA patients (*p* < 0.001, Fig. 3a), but not for *AATK*-variant 2. The *AATK*-*AS* transcripts were not detectable in tissues and cell lines. Our data showed that the levels of DNA methylation and mRNA expression of *AATK* were inversely correlated (*r* = − 0.507, *p* < 0.01, Fig. 3b) suggesting that *AATK*-v1 expression was epigenetically silenced in PDA patients. Furthermore, we extended the analysis of these four methylated probes by validating this finding in a cohort of PDA patients in the TCGA project, from which the follow-up data were available for methylation analysis from the same technology platform. The data showed that these four differentially methylated CpG probes from the TCGA cohort were highly methylated in tumor tissues, consistent with those observed in the NCKU cohort (Fig. 3c). An inverse correlation between *AATK* DNA methylation and mRNA expression was significant in the TCGA dataset (*r* = − 0.411 to − 0.457; Fig. 3d). These data validated that *AATK* DNA methylation and mRNA downregulation are both potential prognostic markers for PDA patients.

### Downregulated AATK protein expression associates with poor overall survival

**Fig. 4:**
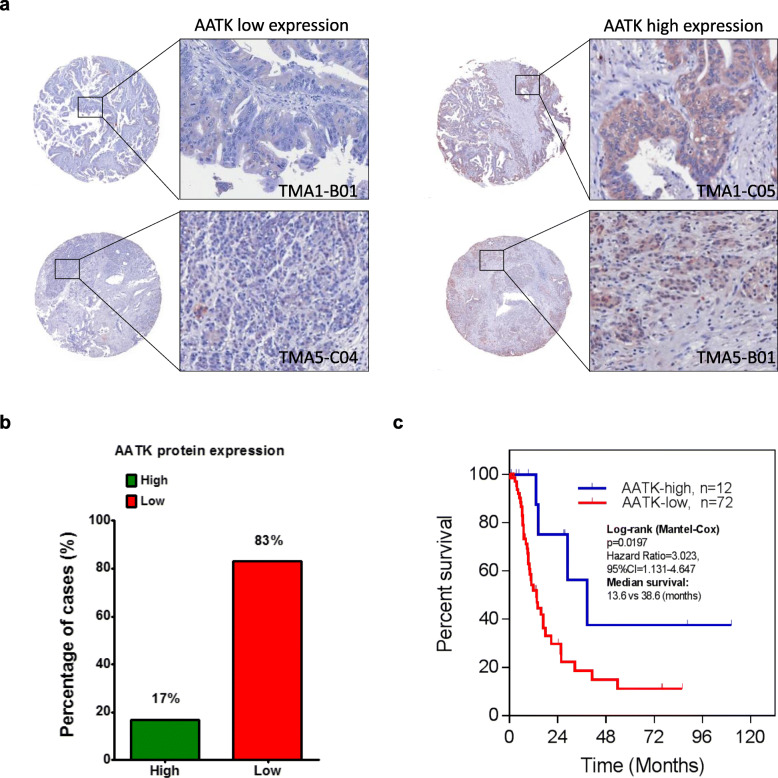
Low protein expression of AATK indicated poorer prognosis in PDA patients. **a** Representative immunohistochemistry (IHC) of AATK protein expression in four PDA patients. **b** AATK protein is expressed at low levels in 83% of PDA patients. **c** Kaplan–Meier curves showing PDA patients with low AATK protein low expression had significantly poorer overall survival than those with high expression. *P* values are shown as indicated

To further elucidate the role of AATK in the clinical outcome of PDA, we evaluated the expression of AATK in 88 PDA and 12 adjacent noncancerous pancreas tissues in a set of tissue arrays by immunohistochemistry (IHC) staining (Table 1). AATK protein expression in PDA patients was then stratified into two groups which are high expression or low expression of AATK. Our data showed that 83% (73/88) of patients expressed a low level of AATK protein in tumor tissues (Fig. 4a, b), and that the differences in patient outcome were significant (*p* < 0.05) in terms of overall patient survival between the two groups. The overall Kaplan–Meier survival curve analysis indicated that patients with low AATK protein expression had significantly worse survival than those with high AATK expression (*p* < 0.05, Fig. 4c). These data suggest the aberrant hypermethylation of AATK is associated with both downregulated mRNA and protein expression in PDA patients. Low AATK expression did not correlate with recurrence (*p* = 0.562) or metastatic status (*p* = 0.395), but was enriched in patients of age > 65 (*p* = 0.055) (Table 1). Therefore, the role and mechanism of AATK in early tumorigenesis of PDA in young patients warrants further investigation.

**Table 1:** Summary of clinicopathological features and follow up of patients with pancreatic cancer

Characteristics	Number (%)	AATK expression		*P*
		Low	High	
Age				
< 65	41 (49.4)	32 (78.0)	9 (22.0)	0.055
> 65	42 (50.6)	39 (92.9)	3 (7.1)	
Sex				
Male	28 (33.7)	23 (82.1)	5 (17.9)	0.530
Female	55 (66.3)	48 (87.3)	7 (12.7)	
Tumor location				
Head	52 (62.7)	43 (82.7)	9 (17.3)	0.142
Neck	6 (7.2)	6 (100.0)	0 (0.0)	
Body	4 (4.8)	2 (50.0)	2 (50.0)	
Tail	6 (7.2)	6 (100.0)	0 (0.0)	
Uncinate	10 (12.0)	10 (100.0)	0 (0.0)	
Others	5 (6.0)	4 (80.0)	1 (20.0)	
Tumor size, cm				
≤ 3	47 (56.6)	40 (85.1)	7 (14.9)	0.897
> 3	36 (43.4)	31 (86.1)	5 (13.9)	
Tumor grade				
Poorly differentiated	17 (20.5)	16 (94.1)	1 (5.9)	0.466
Moderately differentiated	46 (55.4)	39 (84.8)	7 (15.2)	
Well differentiated	20 (24.1)	16 (80.0)	4 (20.0)	
Stage				
I	9 (10.8)	7 (77.8)	2 (22.2)	0.733
II	69 (83.1)	59 (85.5)	10 (14.5)	
III	4 (4.8)	4 (5.6)	0 (0.0)	
IV	1 (1.2)	1 (100.0)	0 (0.0)	
Recurrence status				
Absent	22 (26.5)	18 (81.8)	4 (18.2)	0.562
Present	61 (73.5)	53 (86.9)	8 (13.1)	
Metastatic status				
Absent	39 (47.0)	32 (82.1)	7 (17.9)	0.395
Present	44 (53.0)	39 (88.6)	5 (11.4)	
CA199				
< 37U/ml	14 (16.9)	12 (85.7)	2 (14.3)	0.987
> 37U/ml	63 (75.9)	54 (85.7)	9 (14.3)

### AATK promotes cell apoptosis in PDA cell lines

**Fig. 5:**
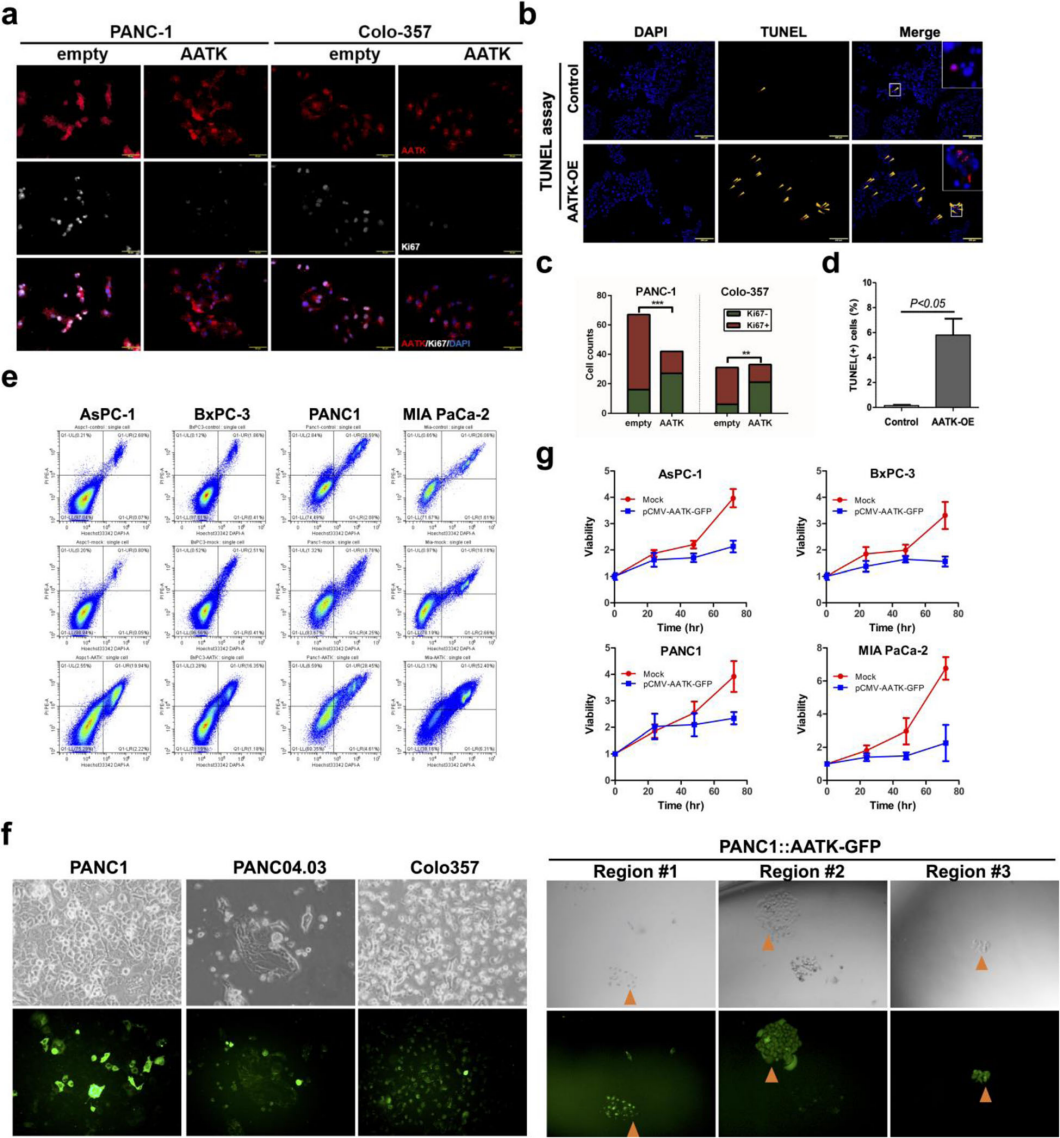
AATK overexpression in pancreatic cancer cell lines increased apoptosis and downregulated proliferation. **a** Apoptosis was detected by the terminal deoxynucleotidyl transferase dUTP nick end labeling (TUNEL) assay in the Panc-04.03 PDA cell line. **b** Staining for proliferation marker Ki67 was performed in cell lines with and without an AATK overexpression plasmid. Statistical assessment was based on a t-test (TUNEL assay) and Fisher’s exact test (cell count of cells positively stained for the proliferation marker by immunofluorescence assay). **c**, **d** Quantification of (**a**) and (**b**), respectively. **e** Cell apoptosis analysis by double staining of propidium iodine and Hoechst 33342 and quantification by flow cytometry. **f** AATK-GFP expression in transient transfection in PANC-1, PANC-04.03, and Colo-357 cell lines (left panel) and in selection for stable AATK-GFP expression clones of PANC-1 cell line. **g** The growth curve of PDA cells with AATK overexpression compared to mock transfection in AsPC-1, BxPC-3, PANC-1, and MIA PaCa-2 PDA cells

Previous studies have shown that AATK overexpression causes apoptosis in melanoma cells [40]. In this study, we independently corroborated whether functional AATK might trigger apoptosis in PDA cell lines. First, AATK was overexpressed in Panc-04.03 cells and the extent of apoptosis by terminal deoxynucleotide transferase nick end labeling (TUNEL) assay was examined. This data showed that Panc-04.03 cells transfected with AATK plasmid DNA increased apoptosis 24 h after transfection (Fig. 5a, c). Next, transient transfection of AATK plasmid DNA decreased cellular proliferation in Panc-1 (p63^-^) and Colo-357 (p63^+^) cell lines, as indicated by Ki67 staining (Fig. 5b, d). This data is in concordance with the apoptosis data. Apoptosis analysis of four pancreatic cancer cell lines with AATK overexpression compared to the empty vector and untransfected control cells showed increased apoptotic cells (Fig. 5e). Stable clonal overexpression of AATK was not achieved due to its pro-apoptotic effects and single clonal cell lines diminished over time (Fig. 5f). Overall, the growth curves for cells with AATK overexpression compared to control in four pancreatic cancer cell lines showed the suppressed static growth rate in four cell lines examined (Fig. 5g). These data showed that AATK overexpression promoted apoptosis and suppressed proliferation in PDA cell lines.

### AATK expression in vivo associates with nuclear VAV1 localization and cell cycle arrest

**Fig. 6:**
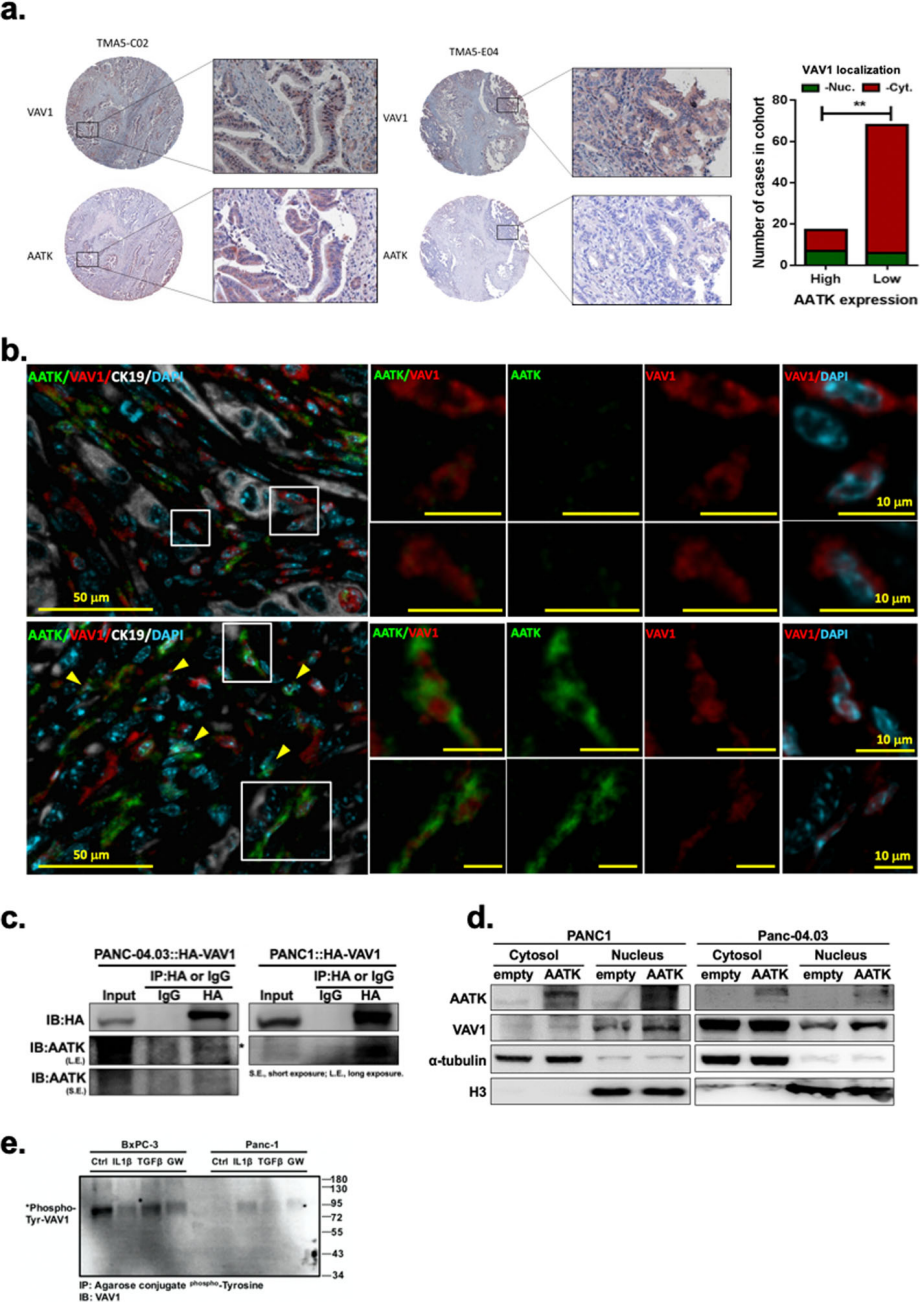
AATK regulated VAV1 cellular localization. **a** AATK is co-expressed with VAV1 in tumor sections of human pancreatic cancer stained with AATK and VAV1 antibodies. Statistical assessment was based on Fisher’s exact test (AATK/VAV1 marker positivity in IHC imaging assays). **b** Tumor sections of KPC2 mice treated with vehicle (upper panel) or 5 mg/kg GW788388 (lower panel) were stained with AATK/VAV1/pan-cytokeratin/DAPI and analyzed by multiple immunofluorescence imaging assays. Representative foci showed that loss of AATK expression in cancer cells co-expresses with cytosolic VAV1 in comparison to those with restored AATK expression after GW788388 treatment were noted with enrichment of nuclear VAV1 expression. Rectangular boxes indicate the region of interest to be magnified in the adjacent panel. Scale bar: 50 μm (left panel) and 10 μm (right panel). AATK, green; VAV1, red; CK19, grey; and DAPI, cyan. **c** Co-immunoprecipitation followed by Western blotting indicated that AATK and VAV1 proteins were incorporated into a protein complex. **d** AATK overexpression increased nuclear VAV1 protein expression. **e** VAV1 tyrosine phosphorylation decreased in response to IL-1β and the TGFβ inhibitor GW788388 but did not decrease in response to TGFβ treatment in Bx-PC3 cells. VAV1 tyrosine phosphorylation were expressed at a low level in Panc-1 PDA cells

To determine the functional role of AATK downregulation in the signaling pathways and networks of QM-PDA, we analyzed a number of them, including squamous cell differentiation, inflammation, hypoxia, metabolic reprogramming, ECM, TGF-β, WNT, MYC, proliferation, autophagy, and RNA processing [12]. Among these, SMAD-dependent cellular responses of the TGFβ signaling pathway have been reported to activate ectopic VAV1 expression via hypomethylation of the VAV1 promoter [43] and regulate tumor progression [44, 45]. Therefore, we examined potential regulation between AATK and VAV1. IHC staining of the same set of human PDA tissue micro-array showed that nuclear localization of VAV1 was significantly associated with AATK expression (Fisher’s exact *t* test, *p* < 0.01) (Fig. 6a). To verify whether this result was consistent in vivo in an animal model, tumor sections of *Kras*^*G12D/+*^; *Trp53*^*flox/flox*^;*Pdx*-*1*-*Cre* (KPC2) mutant mice treated with the TGFβ pathway inhibitor GW788388 or a vehicle control were prepared. In KPC2 mice receiving GW788388 treatment, AATK-positive cells increased in number and were enriched for VAV1 nuclear localization. Conversely, cancer cells in those mice with low or negative AATK expression, cytosolic VAV1 localization was enriched (Fig. 6b). These in vivo findings indicated a potential mechanism for the expression of AATK to regulate the localization of VAV1 protein within the cell nucleus. Next, to determine the interactions between AATK and VAV1 proteins, co-immunoprecipitation experiments were performed with Panc-1 and Panc-04.03 PDA cell lines. This data showed AATK and ectopically transfected VAV1 formed protein complexes in both PDA cell lines tested (Fig. 6c). Overexpression of AATK plasmid DNA increased VAV1 protein levels in the nuclear fraction but not in the cytosolic fraction (Fig. 6d). To determine whether AATK or other proteins might post-translationally modify VAV1 protein, p63^+^ squamous-like BxPC-3 and p63^-^ progenitor-like Panc-1 cells were tested for the tyrosine phosphorylation of VAV1 via Co-IP experiments. The data showed high levels of tyrosine phosphorylated VAV1 in the squamous PDA cell line Bx-PC3, compared to the low levels of phosphorylation in the progenitor-PDA Panc-1 cell line (Fig. 6e). In BxPC-3 cells, phospho^Tyr^-VAV1 showed no change after TGFβ treatment, but it decreased in cells exposed to the TGFβ inhibitor GW788388 (Fig. 6e, left panel). In Panc-1 cells, the phospho^Tyr^-VAV1 level remained low for all treatment groups (Fig. 6e, right panel). Collectively, these results supported that AATK expression was downregulated in KPC2 mice and that inhibition of the TGFβ pathway restored AATK expression, which is concomitant with the nuclear localization of VAV1 protein.

### AATK activation in acinar-to-ductal metaplasia guides ductal epithelial polarization and is silenced during proliferation and progression of PanIN

**Fig. 7:**
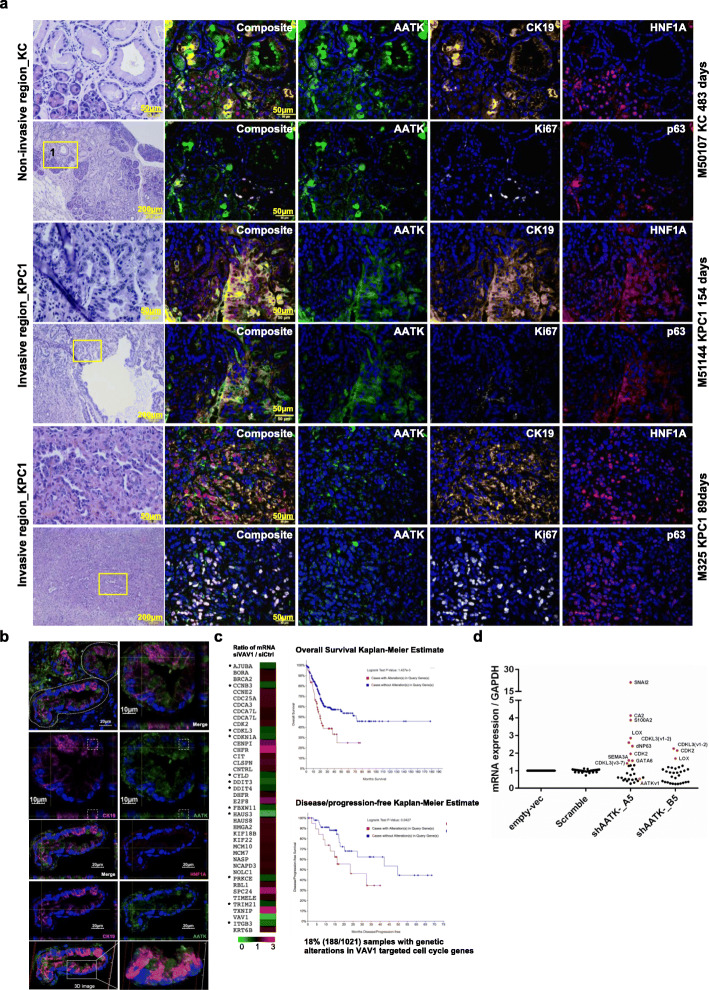
Transient AATK expression in ADM guided cytokeratin 19 intermediate filament and apical-basal polarity. **a** H&E and multiplex immunofluorescence (AATK/CK19/HNF1A/P63/Ki67/DAPI) staining of (**a**) non-invasive pancreatic tissue sections from *Kras*^*G12D/+*^; *Pdx1*-*Cre* mice and invasive ductal sections from *Kras*^*G12D/+*^; *Trp53*^*fl/+*^; *Pdx1*-*Cre* mice. Individual colour channels and merged composite pictures are shown with arrows indicating polarized ADM ductal cells, arrowheads indicating proliferating p63-positive ductal cells, and asterisks indicating HNF1α^+^ acinar cells. **b** Higher magnitude visualization of the cellular localization of AATK, CK19, and HNF1A in the KC mice. **c** VAV1 targeted the cell cycle pathway gene network and cytokeratin 6B expression in Panc-04.03 cells. * AATK mRNA and VAV1 mRNA were positively co-expressed. Kaplan-Meier estimates of the overall survival and disease-free/progression-free survival of patients in the cBioPortal. **d** Relative mRNA expression fold changes in two stable cell lines expressing the AATK shRNA -A5 and -B5 in Mia Paca-2 cells by qPCR. Top upregulated genes were highlighted in red. The mRNA expression were calculated by ∆∆Ct method using GAPDH (circle) as the internal control. Each of the four lentiviral transduced cell pools shAATK-A5, shAATK_B5, Scramble control, and Empty-vector controls were selected by puromycin for 25 days prior to the total RNA isolation. The DNAse digestion, cDNA preparation, and qPCR procedures were conducted with standard protocols

To trace the role of AATK in vivo, serial tissue sections were analyzed by H&E and multiplex immunofluorescence staining. Both the early benign neoplasia, which prior to tumorigenesis, indicates non-cancerous and non-invasive tissue lesion enriched in the *Kras*^*G12D/+*^; *Pdx1*-*Cre* (KC) mice; and the progressive lesions, which are cancerous and invasive neoplasia enriched in the *Kras*^*G12D/+*^; *Trp53*^*fl/+*^; *Pdx1*-*Cre* (KPC1) mice, were analyzed and compared in vivo. Sequential serial sections were stained with AATK and cytokeratin 19 (CK19), HNF1 homeobox A (HNF1α), p63, or Ki67 (Fig. 7a, S2). Based on the imaging analysis, AATK was not expressed in regular acinar clusters (HNF1α^+^) but was upregulated in those transdifferentiating acinar clusters that exhibited diminished acinar HNF1α and increased CK19 co-expression (Fig. 7a, S2). Surprisingly, the protein expression of AATK was in the cytosol of ADM cells and was not colocalized with that of CK19 in the cytoskeleton (Fig. 7b). Instead, AATK localized to the tip of the cytokeratin 19 intermediate filaments in cells characterized with both the acinar and ductal morphology in acinar-to-ductal metaplasia (ADM), and AATK presented with a polarized distribution protruding into the newly formed ductal lumen. Since the turnover of regulated Ki67 protein synchronizes with the cellular proliferative states in S-G2-M phases [46], AATK-positive ADM clusters with distinct Ki67^−^/CK19^+^ expression patterns indicated a non-proliferating G0-G1 cell state for these transdifferentiating ADM cells. However, Ki67 expression was enriched in p63-positive ductal cells. Most of the ductal PanINs were AATK-negative (Fig. 7a, KC). Conversely, in the invasive lesions of the tumor sections of KPC1 mice, the CK19^+^ ductal differentiation pattern was abolished, AATK expression was silenced, CK19 polarity became disoriented, and HNF1A was ectopically expressed in the nucleus of cells but they lacked acinar morphology, clustered acinar architecture, or ductal epithelium continuity (Fig. 7a, KPC1). Additionally, p63 was expressed in cells that are Ki67-positive. In an invasive front of a PanIN lesion with CK19^+^ ductal cells, AATK-positive expression was observed, but the apical-basal polarity was lost (Fig. 7a). HNF1α was ectopically expressed, and p63 was co-expressed in these invasive cells. p63 is activated in proliferating ductal cells and allows transdifferentiation early in ADM and PanIN in the epithelium. Based on non-invasive ADM lesions, transiently activated AATK initiates acinar differentiation into a ductal cell fate with apical-basal polarization in acinar-to-ductal metaplasia. Meta-analysis of our the mRNA expression micro-array data revealed that the cell cycle and mitosis pathway genes CDK2, CDKN1A, DDIT3, E2F8, HAUS3, SPC24, CHFR, CLSPN, TXNIP, and cytokeratin KRT6B might be regulated by VAV1 downregulation [47] (Fig. 7c). The AATK-VAV1-centered gene network highlighted patient outcome in terms of overall survival and disease-free survival of pancreatic cancer. After lentiviral transduction of Mia PaCa-2 cells with AATK shRNA, both the gene knockdown efficiency and gene expression level were determined by qPCR in order to determine cellular differentiation pathways downstream of AATK. Among a panel of gene associates with EMT, squamous PDA, cell cycle, and cellar polarity, our data showed upregulation of CDK2, CDKL3, SNAI2, LOX, CA2, S100A2, ∆Np63, SEMA3A, and GATA6 upon AATK knockdown. These data supported a tumor suppressive role of AATK on cell cycle proliferation, EMT, and squamous differentiation (Fig. 7d). Collectively, AATK was specifically expressed in the ADM foci, but rarely expressed in ductal cells in KC mice. Ductal cellular proliferation and progression was associated with suppressed AATK, activated p63, and induced Ki67 expression. Silenced AATK expression allows the proliferation and progression of ductal PanINs in mutant Kras^G12D/+^ mice.

**Fig. 8:**
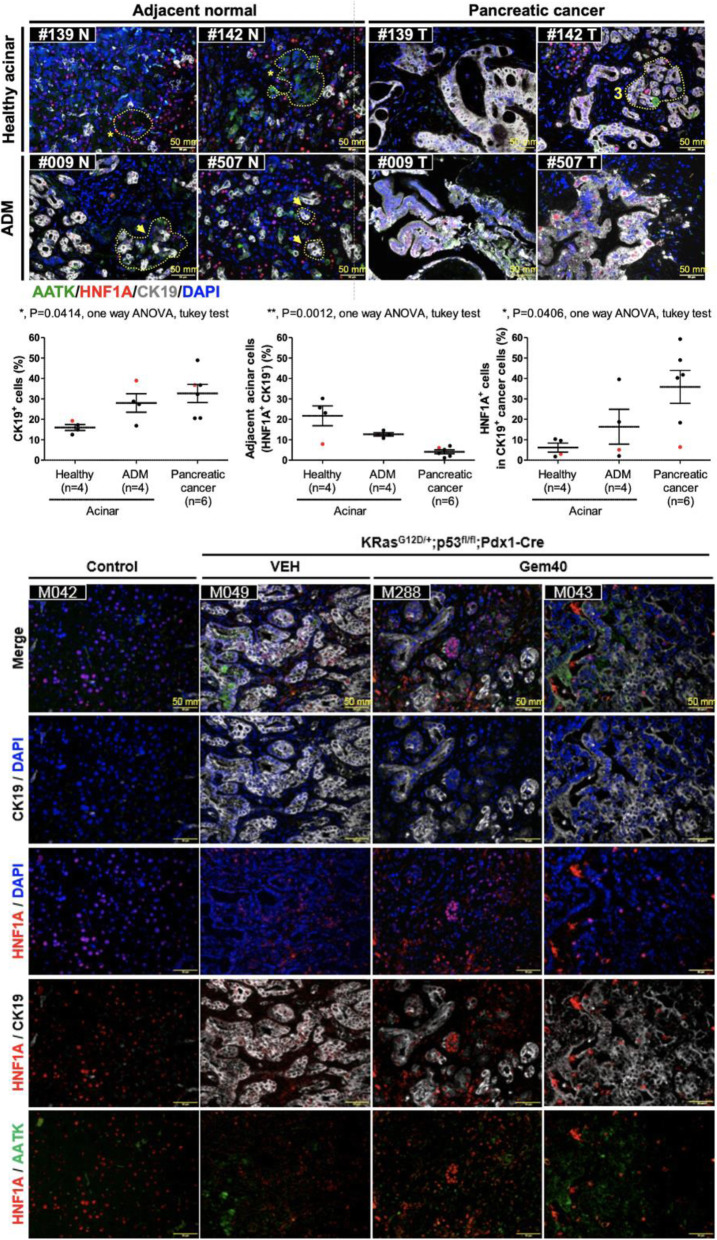
Expression of HNF1A and AATK in human and mouse pancreatic tumors. Analysis of HNF1A and AATK in 6 paired tumor/adjacent normal patient tissue sections (**a**) and in KPC mice received gemcitabine or vehicle control (**b**)

HNF1A is a transcription factor that regulates the differentiation of endodermal genes and regulates the acinar cell identity. Aberrant expression of HNF1A alters lineage differentiation, growth, and the acinar cell plasticity via binding at the enhancers of several acinar genes [48]. HNF1A has been linked to tumor suppressive role based on tumor expression [49, 50] but has been recently identified to promote cancer stemness [16] and resistance to paclitaxel and tyrosine kinase inhibitors [10] in pancreatic tumorigenesis. Analysis of the HNF1A^+^; CK19^+^ cancer cells in local human specimen and in KPC mouse tumors revealed a consistent role of HNF1A expression in cancer progenitor cells. In six pairs of tumor/adjacent normal tissue sections showed positive HNF1A expression in healthy acinar clusters, diminished in ADM, but increased ectopically in ductal cancer cells (Fig. 8a, Fig. S4). In mouse tumors, HNF1A^+^; CK19^+^ cancer cells were present in tumoral tissue sections from both the vehicle-treated and the gemcitabine-treated KPC mice. HNF1A positivity in cancer was consistent with the cancer persister cells, which were not eliminated from the tumor during gemcitabine chemotherapy (Fig. 8b). Together, HNF1A stably expressed in the nucleus of acinar cell clusters and became diminished in ADM followed by induction of AATK expressed in ADM and early PanINs.

Collectively, HNF1α suppresses AATK expression to maintain acinar cell fate in non-invasive lesions in mutant Kras with functional p53, but with the inactivation of p53 in KPC1 mice, ectopic expression of HNF1α led to invasive lesions. AATK and HNF1A in ADM showed an inverse correlation and indicated that HNF1A maintains acinar identity, and AATK expression was induced in ADM to establish apical basal polarity of the ductal epithelium and luminal orientation, upon downregulation of HNF1A in ADM.

## Discussion

To understand the cell fate differentiation during tumorigenesis in the pancreas, we hypothesized that subtype-specific cancer clones should be derived from tissue stem cells and can evolve into intra-tumoral heterogenous clones of cancer-initiating cells. Overall, inverse correlation of *AATK* promoter methylation and the mRNA expression suggested that epigenetic silencing of *AATK* in PDA patients is associated with worse prognosis. Among PDA subtypes, low mRNA expression of AATK associates with a QM-PDA expression signature. Since epigenetic silencing of *AATK* associates with poor overall survival of patients, epigenetic mechanisms might play an important role in the development of QM-PDA. Consistent with this, AATK protein expression significantly associates with nuclear VAV1 localization in PDA patients, in which AATK-negative cases were overrepresented with cytosolic VAV1 localization, epithelial-to-mesenchymal transition, and dissemination of cancer cells. Consistent with reported functions of AATK in neuron [51], we verified that AATK overexpression increases apoptosis, promotes nuclear VAV1 localization, and decreases cellular proliferation both in vitro in PDA cells and in vivo during acinar-to-ductal metaplasia (ADM) in mice. AATK activation in ADM guides ductal epithelial polarization and is silenced during proliferation and progression of PanIN. Aberrant epigenetic silencing of AATK contributes to proliferation in cells in invasive cancer lesions in vivo in KPC mice. Consistent with the role of AATK in neuron to regulate differentiation and apoptosis of their terminally differentiated cells [51], our results suggest that VAV1 interacts with AATK in the course of cell fate commitment and differentiation of pancreatic progenitor cells during tumorigenesis into progenitor cancer clones of PDA.

Acinar-to-ductal metaplasia (ADM) indicates a key transient state of tissue repair. Thus far, ADM studies have indicated that a hallmark of metaplasia is a change in cellular identity that involves a network of lineage-specific transcription factors to respond to cellular and developmental cues [52, 53]. AATK protein may function as a negative regulator of ADM cells preventing development into QM-PDA. During the cell fate transition in ADM, cells respond to a yet unidentified signal or cue presumably for tissue repair in order to create basal-apical polarity and establish ductal cell epithelium after ADM completion. In the current study, it appears that AATK expression is suppressed when the ADM switches into the ductal lineage of non-invasive lesions of KC mice has been accomplished. It remains uncertain about the timing of epigenetic methylation or the chromatin remodeling mechanisms that switch the *AATK* gene on and off in development, in tissue repair, and in tumorigenesis. One possibility is transient suppression by chromatin remodeling once the ductal cell fate is established precisely at the time ADM becomes completed, followed by further DNA methylation of the *AATK* promoter to establish a locked long-term epigenetic trait in the ductal lineage.

AATK protein is a serine/threonine kinase that is highly expressed in neurons, and that suppresses cyclin D5 in axonal outgrowth [38]. AATK-positive cells are enriched for nuclear VAV1 localization, a phenotype that we previously showed maintains better prognosis in tumors as compared to those tumors with cytosolic VAV1 [42]. In Panc-04.03 cells transfected with VAV1 siRNA, gene expression micro-arrays showed decreased mRNA levels of CDKN1A (p21) and upregulated CDK2 expression in the cell cycle pathway (Fig. 7c).

miR-338 is spliced from the AATK intron 7, and therefore, the epigenetic silencing of AATK is consistent with downregulation of miR338-3p via decreased AATK mRNA expression in PDA. Although miR-338 biogenesis results from its host gene, *AATK*, miR-338-3p itself does not target AATK (Table S1). miR338 targets SOX9 mRNA in oligodendrocytes to precisely balance inversely coupled homeostasis between SOX10 and SOX9, in which the transcription factor SOX10 directly transactivates miR338 expression to suppress SOX9 mRNA [54]. SOX 9 is present in not only the progenitors but also the terminally differentiated ductal cells. In support of our finding in the ADM transdifferentiation of acinar cells, recent work showed that SOX9^+^/PTF1A^+^ defines the tip progenitor cells in human fetal pancreas [55], in which *SOX9*; *HNF1A* and *PTF1A*; and *PDX1* encode the key lineage transcription factors for the pancreatic progenitor, acinar, and ductal lineages, respectively. It remains unclear whether the *AATK* gene guards the cue for properly conducted ADM for precise tissue repair by simultaneous usage of biogenesis into miR-338-3p to suppress SOX9 during which AATK’s protein function is used to guide basal-apical polarity, and then followed by the epigenetic mechanism of gene silencing as soon as ADM is complete. Further follow-up studies should address the validity of this model.

Both the acinar differentiation and the oligodendrocyte differentiation pathways are the top functional pathways we identified when we inactivated VAV1 by siRNA transfection in our previous report. The functional outcome of miR-338 in regulating the SOX10-miR-338-SOX9 axis in oligodendrocytes is consistent with the suppression of SOX9 in acinar cell differentiation from the bi-potent progenitor lineage in pancreas development [56]. Downregulation of miR-338 has been identified in the *BRAF*^*V600*^ mutation in melanoma [57], but the mechanism remains unclear. In support of the tumor suppressive role of miR-338-3p, it has been reported that SOX9 regulates ERBB2 expression in PDA [58]. To estimate the expression of miR-338-3p in pancreatic tumorigenesis, we found that miR338-3p is downregulated in our in situ hybridization analysis in 4 PDA tumor sections (Fig. S3). Although the data are not sufficient at the moment, the molecular mechanism of regulation of VAV1, AATK, and miR338-3p and their roles in in the regulation of early oncogenic alterations on lineage progenitor cells of pancreas development should be further studied.

The *AATK* promoter is hypermethylated in several epithelial cancer entities, including the lung, breast, skin, cervix, larynx, and pancreas. Overexpression of AATK inhibits the growth of lung cancer and cervix cancer cells [41] and is involved in apoptosis in melanoma [40]. Although previous reports of AATK gene function extensively revealed the pro-apoptosis effects in solid tumors and axon-guidance in neurons, our results are the first to suggest that AATK is essentially and sufficiently involved in cellular growth and cell lineage transdifferentiation in the G0-G1 cell cycle. Transdifferentiating HNF1α^+^ acinar cells gradually downregulate HNF1α and initiate AATK expression in vivo in ADM without inducting apoptosis to destroy the acinar cells. Consistent with a previous report [16], ectopic expression of HNF1α and p63 in invasive cancer (Fig. 7a, KPC1) further suggested a role for HNF1α and p63 in oncogenic transformation and molecular subtype stratification.

Our work identified that HNF1A expression is diminished during ADM. Ectopic expression of HNF1A in CK19^+^ cancer cells might indicate multistep tumorigenesis of cancer clones comprised of a HNF1A^+^ lineage that might lead to cancer subtypes. Underlying factors that ectopically activates HNF1A in cancer cells represents an important event in aberrant transdifferentiation in ADM and in EMT. Further studies such as lineage tracing experiments to verify whether the founding cancer clones arises early as in the acinar cell lineage via ADM transdifferentiation into the tissue origin of cancer are needed. The functional mechanism by which HNF1A regulates AATK signaling in the expression of acinar, ductal, and mesenchymal lineages warrants further investigation.

## Conclusions

Epigenetic silencing of AATK represents a novel biomarker for subtype stratification and survival prognosis in QM-PDA. To our knowledge, this study reports for the first time that *AATK* is indeed downregulated in PDA patients due to a frequently inactivated *AATK* promoter by hypermethylation, and it complements previous studies on subtype gene signatures of PDA [2]. Upon in vivo transdifferentiation cues to trigger ADM, AATK is induced essentially for transdifferentiating HNF1α^+^ acinar cells to establish both a ductal cell fate and apical-basal polarity as soon as the co-expression of SOX9 in the acinar cells is established to form the SOX9^+^/PTF1α^+^ tip progenitor cells. In non-invasive tissue foci, downregulation of HNF1α in acinar cells during ADM associates with AATK expression to suppress cell cycle progression, instead of triggering apoptosis to destroy acinar cells. When HNF1α and p63 are ectopically expressed in the invasive legions of KPC mice with p53 inactivation, AATK is functionally disrupted, cannot control apical-basal polarity, and becomes downregulated to relieve cell cycle suppression. The effect of AATK-VAV1 signaling on the cell cycle, proliferation, and cellular transdifferentiation is critical in cell cycle suppression in pancreatic tissue repair when p53 is intact. Loss of AATK expression when p53 is inactivated leads to cell cycle proliferation and Ki67 expression, concomitant with ectopic expression of HNF1α and p63. The molecular mechanisms by which AATK affect the underlying subtype of transdifferentiation warrant further investigation, and further studies are needed to improve our understanding of the cell origin and clonal interactions of pancreatic cancer subtypes.

## Materials and methods

### Clinical specimen

In this study, two sets of PDA samples were recruited. The first set, containing 61 cases of PDA samples (48 tumor tissues and 13 adjacent noncancerous tissues), was used for the methylation analysis of AATK CpG sites by EpiTYPER. The second set, 94 PDA samples on a tissue microarray, was used for the analysis of AATK protein expression by immuno-histochemical staining and its correlation with patients’ prognosis.

### EpiTYPER high throughput DNA methylation measurement

The quantitative DNA methylation analysis was conducted as previously reported. In brief, EpiTYPER biochemistry starts with bisulfite treatment of 1 μg of high-quality genomic DNA according to standard protocol using EZ-DNA methylation kit (Zymo Research), followed by PCR amplification of target regions and then detection by MALDI-TOF mass spectrometry (Agena biosciences).

### Animal model

To verify the localization of AATK in spontaneous progression of PDA in vivo, the well characterized *LSL*-*Kras*^*G12D/+*^; *Trp53*^*flox/flox*^; *Pdx1*-*Cre* (KPC) mice with spontaneous tumors at the age of 4, 6, and 8 weeks were sacrificed and the pancreatic tissue were harvested and dissected into three parts, each processed for formalin-fixed paraffin-embedded (FFPE) and for RNA later preservation (QIAGEN) tissue histological analysis.

### Immunohistochemistry (IHC) staining and scoring

The protein expression level of AATK was evaluated by IHC of tumor tissues from tissue micro-array (TMA) of PDA patients as described in previous report [59, 60]. Paraffin blocks of tumors were cut into 5 μm slices and then processed using standard deparaffinization-rehydration techniques. The evaluation of the IHC was conducted blindly without knowledge of the clinical and pathologic characteristics of the patients. The surrounding non-neoplastic stroma served as an internal control for each slide. All scores were determined in a blinded manner by senior pathologists. Scoring was conducted according to the ratio and intensity of positive-staining cancer cells and scored as 0–3. The final score was designated as low or high expression group as follows: score 0–1, low expression; score 2–3, high expression.

### Multiplex immunofluorescence imaging

Pancreas/PDA collected from mice were fixed in 10% formalin and embedded in paraffin. Serial sections of 5-μm-thick were prepared for the H&E stain and the immunohistochemistry staining as per manufacturer’s instructions. Briefly, after deparaffinization, rehydration, antigen retrieval in heated citric acid buffer (pH 6.0), and blocking with antibody diluent, the sections were immersed with primary antibody and followed with corresponding secondary horseradish peroxidase (HRP) antibody. Tyramide signal amplification (TSA) with different fluorophore interacted with HRP by the covalent binding to label the signals of the primary antibody. Additional antigen retrieval in heated citric acid buffer (pH 6.0) removed the bounded antibodies before the second round of next primary antibody staining. After three sequential reactions, all sections were mounted with mounting medium with DAPI and examined on the light microscopy by using an Olympus Model BX53 with a DP80 microscope digital camera (Olympus Corp., Tokyo, Japan). For PDA cell lines, the cells were plated at the density of 2 × 10^4^/cm^2^ in 6-well plate. After 48 h of transfection, cells were trypsinized and plated on a coverslip in a 6-well plate. Staining was performed by immunofluorescence using the Opal 4-color lHC kit (Perkin Elmer). The cell were subsequently stained with species-matched secondary antibodies. Antibodies and dilution factors were listed in supplement table 1.

### Cell culture

Human PDA cell lines AsPC-1, BxPC-3, MiaPaCa-2, Panc-1, and Panc-04.03 were purchased from ATCC (Manassas, VA, USA). AsPC-1, BxPC-3, Panc-04.03, and Colo-357 cells were maintained with RPMI-1640 medium. MiaPaCa-2 and Panc-1 cells were maintained with Dulbecco’s modified Eagle’s medium. HPDE cells was maintained with defined keratinocyte serum–free medium (keratinocyte-SMF, Gibco), and the defined Keratinocyte-SFM Growth Supplements, including the bovine pituitary extract (BPE), epidermal growth factor (EGF), and 1% penicillin-streptomycin (P/S, Caisson Labs) to the final concentration of 100 units and 100 μg, respectively. Both the RPMI and DMEM medium were supplemented with 10% fetal bovine serum (FBS, Gibco), and 1% P/S. Panc-04.03 cell culture medium was additionally supplemented with 20 units/ml human recombinant insulin (Sigma). All cells were cultured at 37 °C in a 5% CO_2_ chamber.

### AATK siRNA and transfection

Cells at the density of 2 × 10^4^/cm^2^ (MiaPaCa-2) and 1 × 10^4^ cm^2^ for Panc-1 were plated in 2 ml antibiotics-free medium in 6-well plate to reach 40–50% confluency at the time of transfection. A pooled mixture of four siRNA oligonucleotides targeting AATK expression (Dharmacon, supplementary Table S2) was used for transfections with Lipofectamine 2000 (Invitrogen) according to the manufacturer’s protocol at the concentration of 50 nM. The resulting cells were collected after transfection for 48 h.

### MTT assay

For the cell viability assay with MTT, 5 × 10^3^ cells in 100 μl culture medium were seeded per well in 96-well plates. At each time point after transfection, MTT reagents were added into wells. Cells were incubated at 37 °C in a CO_2_ incubator for 2 h. The supernatant was removed and 100 μl dimethyl sulfoxide was added to each well before measuring the OD_570_ by ELISA reader. Each assay was repeated in at least three to six replicates.

### Quantitative reverse transcriptase real-time PCR (qRT-PCR)

Total RNA was isolated from cells with the AllPrep DNA/RNA/miRNA Universal Kit (QIAGEN) according to the manufacturer’s protocol. A total of 2 μg RNA was reverse-transcribed into cDNA using M-MLV Reverse Transcriptase (Promega). Primers were synthesized by IDT at standard desalting purity. Universal Probe Library (UPL, Roche) based PCR assays for mRNA expression were performed via qPCR using the Light Cycler 480 detection system (Roche Life Science) with GAPDH as internal control. For each assay, a mixture of 8 μl containing each of 4 μL 2× UPL Master Mix, 0.4 μl 10 μM qPCR forward and reverse primers, 0.1 μl UPL probe, 1.5 μl RNase-free water, and 2 μl cDNA were prepared in each well in a 384-well plate. The plate was sealed, spun-down to collect the reaction components at the bottom, and transferred to the LightCycler 480 instrument (Roche). The PCR programs were as follows: initial heat activation at 95 °C for 10 min; three step cycling of 45 cycles of denaturation at 95 °C for 10 s, annealing at 60 °C for 30 s, extension at 72 °C for 1 s; and then heating to 95 °C for 1 min followed by cooling to 40 °C for 2 min, and a final heating step to 95 °C and keep in 40 °C.

### Terminal deoxy transferase dUTP nick end labeling assay

Cultured cells were fixed by 4% paraformaldehyde (PFA). All the stain procedures followed the manufacturer’s protocol (Roche in situ Cell Death Detection Kit with Fluorescein label from MERCK Sigma, USA). Briefly, fixed cells were permeabilized with 0.1% Triton X-100 in 0.1% sodium citrate buffer for 8 min. After PBS washing, the cells were incubated in terminal deoxynucleotide transferase nick end labeling (TUNEL) reaction mixture containing terminal deoxynucleotidyl transferase and fluorescein labeled nucleotide mixture for 60 min at 37 °C in dark. Mounting medium supplemented with DAPI was used to cover the labeled cells for detection of fluorescence signals under microscope with an Olympus Model BX53 with a DP80 microscope digital camera (Olympus Corp., Tokyo, Japan).

### Extraction of cytoplasmic and nuclear protein

After washing twice in cold PBS, cells were resuspended in hypotonic lysis buffer (20 mM HEPES pH 7.4, 2 mM MgCl2, 10 mM KCl, 1 m M DTT, 0.05% NP-40, and protease Inhibitor (Roche)) and incubated on ice for 10 min. Vortex gently for 5 s to lyse cells. The cell lysate was centrifuged at 800 g for 5 min at 4 °C. The supernatant was transferred into a new tube and centrifuged again at 11,000 g for 20 min at 4 °C to deplete the membrane and mitochondria debris. The collected supernatant was the cytosolic fraction. The nuclei pellet from cell lysate was washed three times in cold PBS and the nuclear protein was extracted in RIPA buffer (50 mM Tris-HCl pH 7.4, 150 mM NaCl, 1% NP-40, 0.25% Sodium deoxycholate, 1 mM EDTA) on ice for 10 min. The nuclear lysate on ice was homogenized by sonication with 2 × 6 s at output 7 W with a 1-min break between sonication cycles. Finally, the nuclear extraction was centrifuged at 11,000 g for 20 min at 4 °C and collected the supernatant as the nuclear fraction.

### Immunoprecipitation

Immunoprecipitation was performed by using Dynabeads (10003D, Dynabeads™ Protein G, Thermo Fisher Scientific Invitrogen). According to the manufacturer’s instructions, the Dynabeads were pre-incubated with antibodies at room temperature for 10 min and mixed with protein lysate with rotation at 4 °C for 60 min. Dynabeads were separated by magnet and washed with PBST (1× PBS pH 7.4, 0.02% Tween 20). The immunoprecipitated protein was eluted after adding SDS sample buffer at 95 °C for 10 min for Western blot directly or stored at − 20 °C.

### Lentiviral transduction

Two AATK shRNA constructs and two non-targeting controls of the scramble and the empty vector were individually delivered to Mia PaCa-2 PDA cells via lentiviral transduction followed by puromycin selection for 25 days to generate a pool of polyclonal cancer cells with stable expression of the constructs. In both polyclonal cell lines that express the A5 and B5 AATK shRNA constructs, the mRNA expression was analyzed by qPCR.

### Statistical analyses

Each CpG unit was analyzed. Quantitative DNA methylation median levels were compared between tumor and normal samples using the nonparametric Mann–Whitney test for unpaired observations. The association between categorical clinical-pathological factors and cluster assignment was tested with Fisher’s exact test. The distributions of overall survival and recurrence-free survival were estimated using the Kaplan–Meier method. Clusters were tested for differences in recurrence-free survival using the log-rank test. Correlation of methylation levels between CpG sites, and between methylation and protein expression, were assessed using Spearman’s rank correlation coefficient. All tests are two-sided. All analyses were performed using the statistical software GraphPad Prism (GraphPad Software, CA USA) Version 6.07.

## Supplementary information

**Additional file 1: Figure S1.** The TCGA cohort patients formed clusters of: (1) benign (n=36), (2) early development and differentiation (n=86), (3) transition (n=36), and (4) QM-PDA (n=20) based on differential gene expression patterns.

**Additional file 2: Figure S2.** Collection of AATK expression in ADM in KC mice. Insert, higher magnification of Fig. 7a, region 1.

**Additional file 3: Figure S3.** miR-338-3p in situ hybridization in adjacent normal and tumoral sections of pancreatic cancer. U6 snRNA expression was used as positive control.

**Additional file 4: Figure S4.** The individual features of human pancreatic cancer and adjacent normal tissue sections.

**Additional file 5: Figure S5.** Schematic illustration of pancreatic acinar cell in acinar-to-ductal metaplasia and tumorigenesis into poorly differentiated pancreatic cancer subtype. In the acinar-to-ductal metaplasia (ADM), acinar cells display a high level of plasticity and they can transdifferentiate to a progenitor-like ductal cells. Additionally, when pancreas suffer from injury, this reversible mechanism is to repair the tissue. Genes that guard the cellular apical-basal polarization in response to a yet unidentified signal or cue may control the architecture of lumen formation and synchronization of cellular cooperation. Reciprocal or transient epigenetic mechanism of progenitor cells or acinar cells might lead to cell differentiation and post-mitotic state epigenome in quasi-mesenchymal PDA.

**Additional file 6: Table S1.** Survival analysis of VAV1 targeted the cell cycle pathway gene network and cytokeratin 6B expression in a combined analysis of 1207 pancreatic cancer samples from 10 studies included in the cBioPortal [61, 62].

**Additional file 7: Table S2.** Details of reagents and materials. #, not available; *, in the TSA IHC Kit a higher dilution factor was chosen for optimized signal to background ratio.

## Data Availability

The datasets supporting the conclusions of this article are included within the article and its additional files. The datasets used and analyzed during the current study are available from the corresponding author on reasonable request (please contact Dr. Po-Hsien Huang, email: phhuang@mail.ncku.edu.tw). The data generated by the TCGA research network have been publicly available by the TCGA consortium under https://portal.gdc.cancer.gov/ and https://cancergenome.nih.gov/.

## Abbreviations

FFPE: Formalin-fixed paraffin-embedded
IHC: Immunohistochemistry
ISH: In situ hybridization
TMA: Tissue micro-array
AATK: Apoptosis-associated tyrosine kinase
PDA: Pancreatic ductal adenocarcinoma
QM-PDA: Quasi-mesenchymal PDA
