# Supplementary figures and images for: Epigenetic silencing of AATK in acinar to ductal metaplasia in murine model of pancreatic cancer
Source: Clin Epigenetics. 2020 Jun 17;12:87. doi: 10.1186/s13148-020-00878-6 (PMC7301993; doi:10.1186/s13148-020-00878-6)

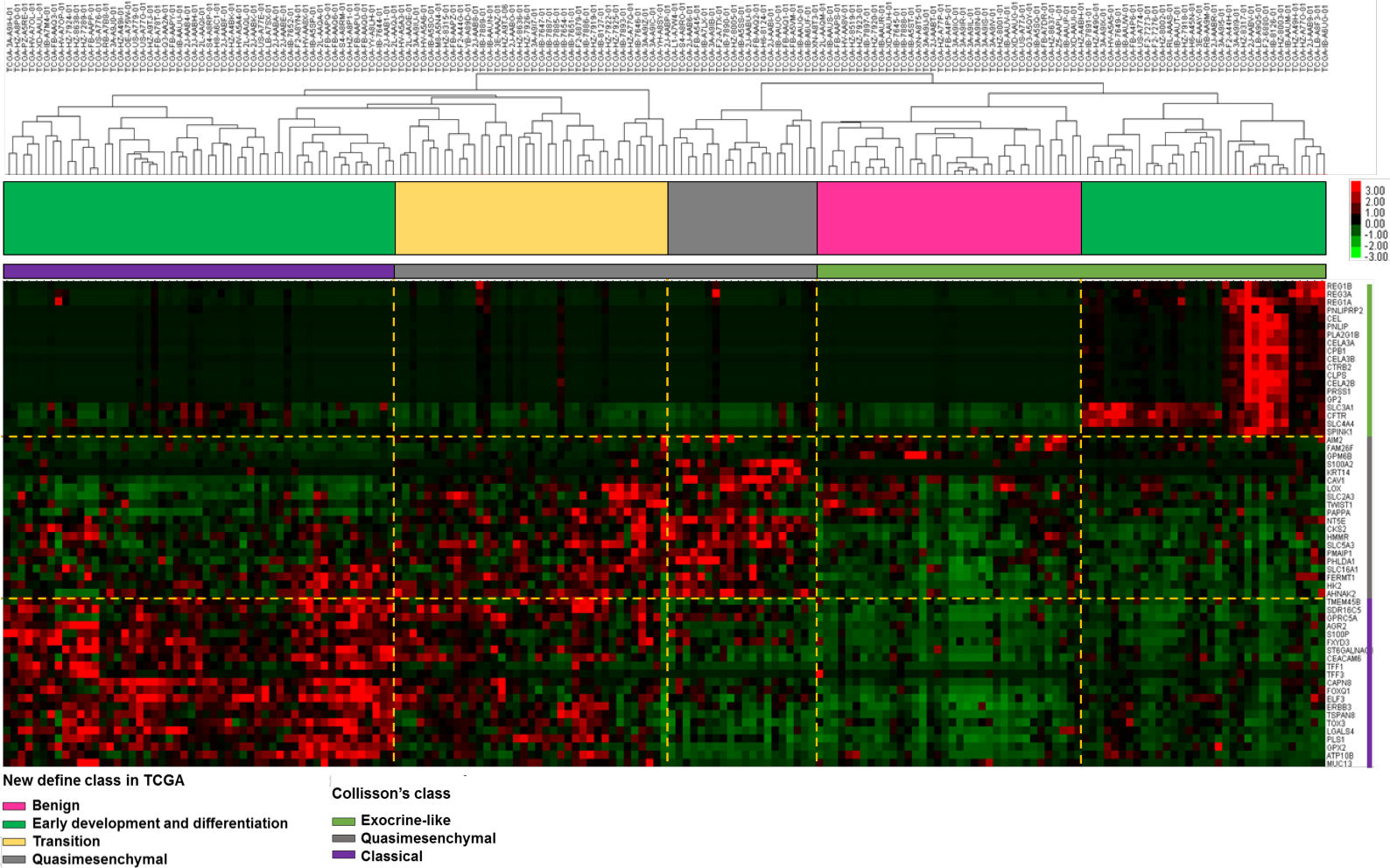

Supplement: Supplementary file 1 — Additional file 1: Figure S1. The TCGA cohort patients formed clusters of: (1) benign (n=36), (2) early development and differentiation (n=86), (3) transition (n=36), and (4) QM-PDA (n=20) based on differential gene expression patterns. [file 13148_2020_878_MOESM1_ESM.docx]

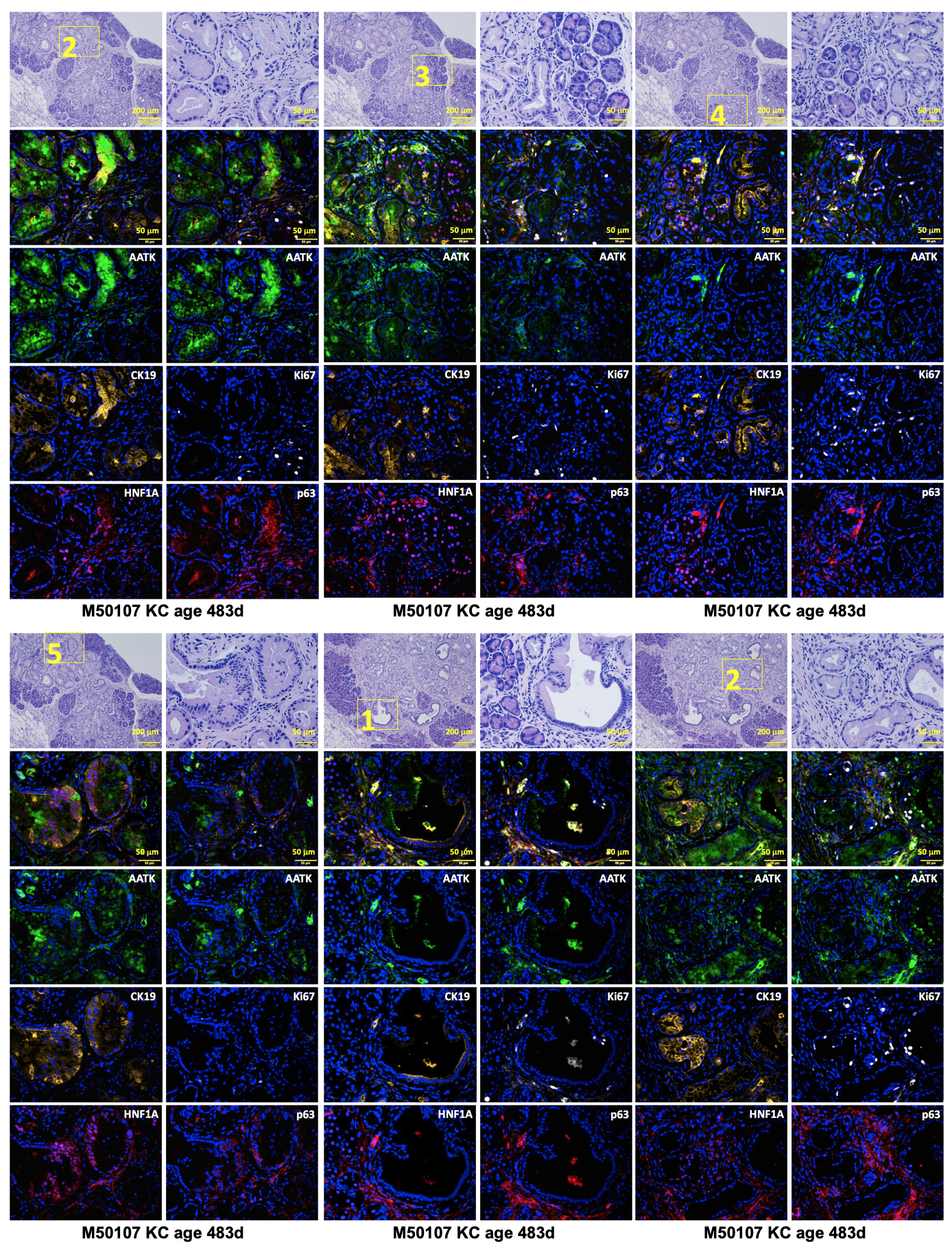

Supplement: Supplementary file 2 — Additional file 2: Figure S2. Collection of AATK expression in ADM in KC mice. Insert, higher magnification of Fig. 7a, region 1. [file 13148_2020_878_MOESM2_ESM.docx]

**
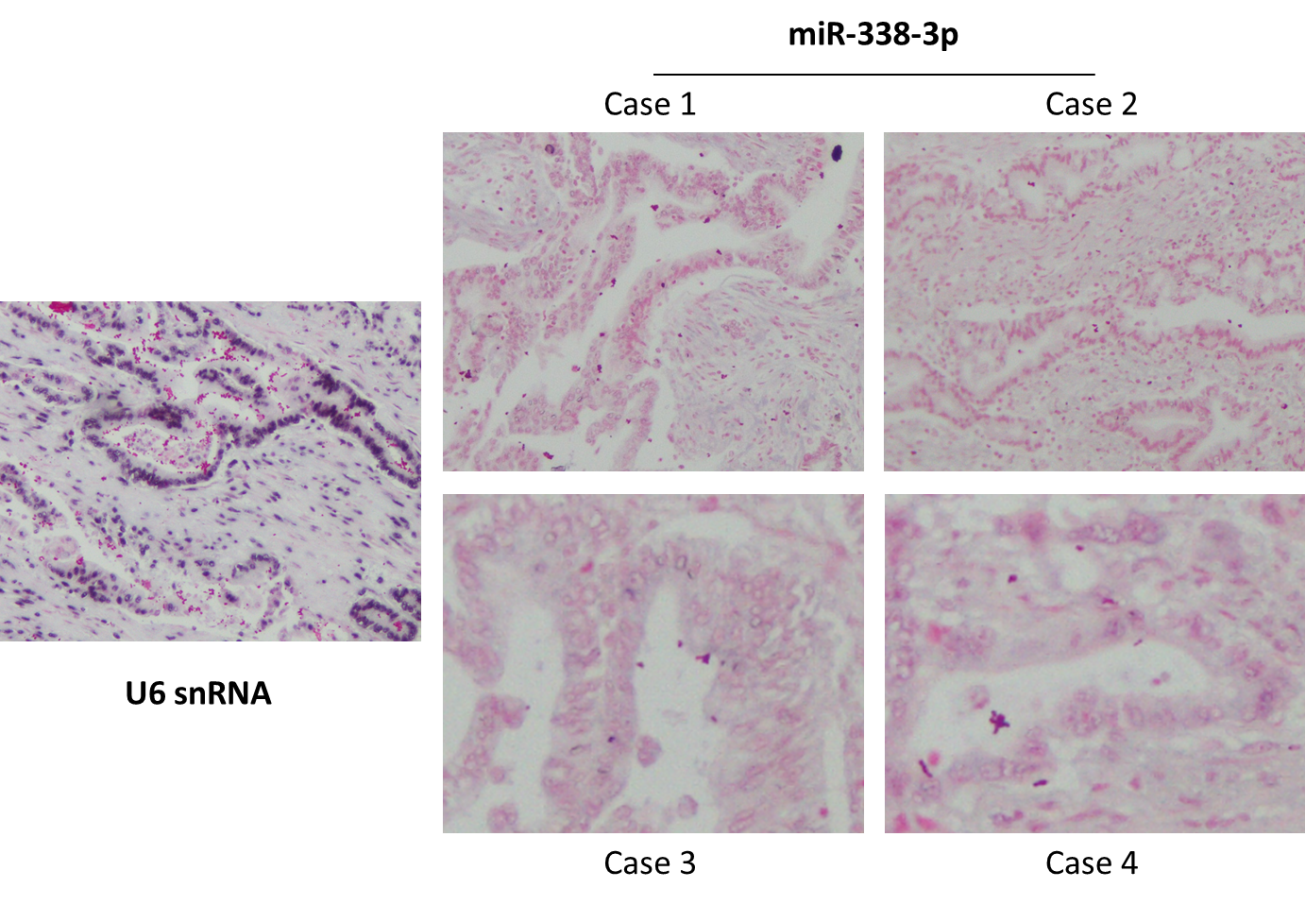
**

Supplement: Supplementary file 3 — Additional file 3: Figure S3. miR-338-3p in situ hybridization in adjacent normal and tumoral sections of pancreatic cancer. U6 snRNA expression was used as positive control. [file 13148_2020_878_MOESM3_ESM.docx]

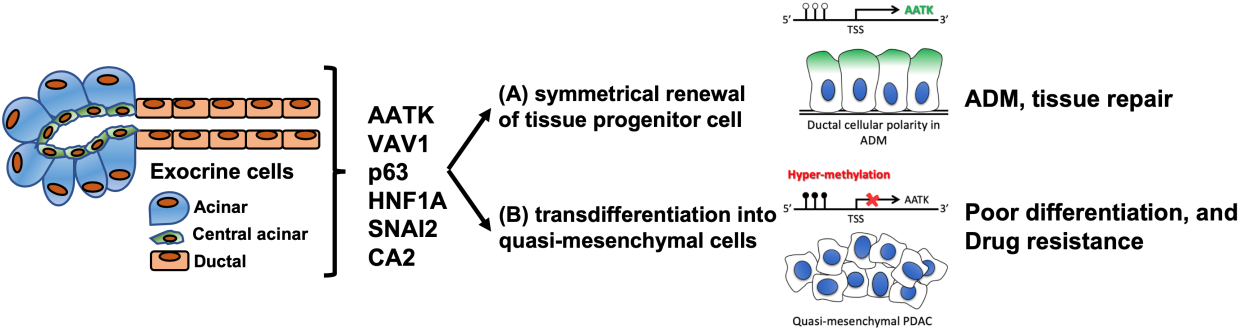

Supplement: Supplementary file 5 — Additional file 5: Figure S5. Schematic illustration of pancreatic acinar cell in acinar-to-ductal metaplasia and tumorigenesis into poorly differentiated pancreatic cancer subtype. In the acinar-to-ductal metaplasia (ADM), acinar cells display a high level of plasticity and they can transdifferentiate to a progenitor-like ductal cells. Additionally, when pancreas suffer from injury, this reversible mechanism is to repair the tissue. Genes that guard the cellular apical-basal polarization in response to a yet unidentified signal or cue may control the architecture of lumen formation and synchronization of cellular cooperation. Reciprocal or transient epigenetic mechanism of progenitor cells or acinar cells might lead to cell differentiation and post-mitotic state epigenome in quasi-mesenchymal PDA. [file 13148_2020_878_MOESM5_ESM.docx]
