# Supplementary material for: Epigenetic silencing of AATK in acinar to ductal metaplasia in murine model of pancreatic cancer
Source: Clin Epigenetics. 2020 Jun 17;12:87. doi: 10.1186/s13148-020-00878-6 (PMC7301993; doi:10.1186/s13148-020-00878-6)
Supplement: Supplementary file 6 — Additional file 6: Table S1. Survival analysis of VAV1 targeted the cell cycle pathway gene network and cytokeratin 6B expression in a combined analysis of 1207 pancreatic cancer samples from 10 studies included in the cBioPortal [61, 62]. [file 13148_2020_878_MOESM6_ESM.docx]

|  | Number of cases, total | Number of cases, deceased | Median months survival | Number of cases, total | Number of cases, relapsed/progressed | Median months disease-free |
| --- | --- | --- | --- | --- | --- | --- |
| Cases with Alterations in query genes | 62 | 35 | 19.82 | 20 | 10 | 23.53 |
| Cases without Alterations in query genes | 219 | 79 | 71.73 | 49 | 13 | 49.67 |
