# Supplementary material for: Epigenetic silencing of AATK in acinar to ductal metaplasia in murine model of pancreatic cancer
Source: Clin Epigenetics. 2020 Jun 17;12:87. doi: 10.1186/s13148-020-00878-6 (PMC7301993; doi:10.1186/s13148-020-00878-6)
Supplement: Supplementary file 7 — Additional file 7: Table S2. Details of reagents and materials. #, not available; *, in the TSA IHC Kit a higher dilution factor was chosen for optimized signal to background ratio. [file 13148_2020_878_MOESM7_ESM.docx]

| **Antibodies** | **Source** | **Identifier** | **RRID** | **Dilution ratio*** | **Comment** |
| --- | --- | --- | --- | --- | --- |
| AATK | Sigma-Aldrich clone 5B8 | WH0009625M3 | AB_1839265 | 1:1300 |  |
| AATK | Santa Cruz Biotechnology | sc-100436 | AB_2219997 | 1:1300 | Target protein |
| AATK | GeneTex | GTX16550 | N/A | 1:200 (WB) | Target protein |
| Cytokeratin | Agilent | M3515 | AB_2132885 | 1:500 | Tumor epithelial cells |
| HNF1A | Abcam | ab204306 | N/A^#^ | 1:2500 | Transcription factor |
| Ki67 | GTX | GTX16667 | AB_422351 | 1:1000 | Cells proliferation |
| p63 | Cell Signaling Technology | 39692 | AB_2799159 | 1:5000 | The squamous cells |
| VAV1 | Millipore Sigma | HPA001864 | AB_1858722 | 1:500 | Nucleus / Cytosol |
| **Cell culture** |  |  |  |  |  |
| AsPC-1 | ATCC | CRL-1682 | CVCL_0152 | RPMI | 10% FBS, 1% P/S |
| BxPC-3 | ATCC | CRL-1687 | CVCL_0186 | RPMI | 10% FBS, 1% P/S |
| MIA PaCa-2 | ATCC | CRL-1420 | CVCL_0428 | DMEM | 10% FBS, 1% P/S |
| Panc1 | ATCC | CRL-1469 | CVCL_0480 | DMEM | 10% FBS, 1% P/S |
| Panc-04.03 | ATCC | CRL-2555 | CVCL_1636 | RPMI | 10% FBS, 1% P/S, 20 Units/ml human recombinant insulin |
| Fetal Bovine Serum (FBS) | Thermo Fisher Scientific Gibco | 10437028 |  |  |  |
| RPMI 1640 Medium | Thermo Fisher Scientific Gibco | 23400021 |  |  |  |
| DMEM | Thermo Fisher Scientific Gibco | 12800017 |  |  |  |
| Defined Keratinocyte-SFM (1X) | Thermo Fisher Scientific Gibco | 10744019 |  |  |  |
| Insulin, Human Recombinant | MERCK Sigma | 91077C |  |  |  |
| Epidermal Growth Factor (EGF) | MERCK Sigma | E4127 |  |  |  |
| **Chemicals and reagents** | **Source** | **Identifier** | **RRID** |  |  |
| Lipofectamine 2000 | Thermo Fisher Scientific Invitrogen | 11668019 |  |  |  |
| MTS assay kit |  |  |  |  |  |
| Thiazolyl Blue Tetrazolium Blue (MTT) | MERCK Sigma | M5655 |  |  |  |
| RNALater RNA Stabilization Reagent | Qiagen | 76106 |  |  |  |
| GW788388 | MedchemExpress Inc. |  |  |  |  |
| Dynabeads™ Protein G for Immunoprecipitation | Thermo Fisher Scientific Invitrogen | 1003D |  |  |  |
| Protease inhibitor | MERCK Sigma Roche | 4693132001 |  |  |  |
| M-MLV Reverse Transcriptase | Promega | RM1705 |  |  |  |
| LightCycler 480 Probes Master | Roche Life Science | 4887301001 |  |  |  |
| Universal ProbeLibrary Set, Human | MERCK Roche | 4683633001 |  |  |  |
| EZ-DNA methylation kit | Zymo Research | D5003 |  |  |  |
| EpiTect Whole Bisulfitome Kit | Qiagen |  |  |  |  |
| AllPrep DNA/RNA/miRNA Universal Kit | Qiagen |  |  |  |  |
| ZR-Duet™ DNA/RNA MiniPrep | Zymo Research | D7001 |  |  |  |
| TSA 4-color Manual IHC Kit | PerkinElmer, USA | PK-NEL810, |  |  |  |
| TUNEL assay by In Situ Cell Death Detection Kit, Fluorescein | MERCK Sigma Roche | 11684795910 |  |  |  |
| TUNEL assay by In Situ Cell Death Detection Kit, | Roche life science | 11684795910 |  |  |  |
| Fluorescein | MERCK Sigma, USA |  |  |  |  |
| **Plasmids sequences and primer assays** | **Source** | **Identifier** | **RRID** |  | **Sequence** |
| AATK () Human Tagged ORF Clone (AATK-GFP) | Origene | RG219135 |  |  | NM_001080395 |
| AATK siRNA mix pool of 4 | Dharmacon |  |  |  | Mix pool of four sequences 1, 2, 3, and 4. |
| UPL human probe set | Roche life science |  |  |  |  |
| UPL master mix | Roche life science |  |  |  |  |
| GAPDH_F/R | IDT |  |  |  | F; AGCCACATCGCTCAGACAC |
|  |  |  |  |  | R; GCCCAATACGACCAAATCC |
| AATK-v1_F/R | IDT |  |  |  | F: CACCAGGTGAAGGTCCAAG  R: CCCTCCGCATTCTCAAAC |
| AATK-v2_F/R | IDT |  |  |  | F: CTTCAGCTCGCACTTCGAC |
|  |  |  |  |  | R: AAGAGCCCGGAGAAAGACA |
| AATK-AS_F/R | IDT |  |  |  | F: TTCCAGAACTCTCGAAAGAAGC |
|  |  |  |  |  | R: GGGCCAGAGCAGACGTAG |
| AATK-v1-201F/R | IDT |  |  |  | F: TGCCTGTAAGAAGGGCGGTAT |
|  |  |  |  |  | R: CACGTCTGTGGACTTGAGGA |
| AATK-v2-148F/R | IDT |  |  |  | F: ACCAGGTGAAGGTCCAAGG |
|  |  |  |  |  | R: GTGAGTGGCAGGACGTACAC |
