# Supplementary material for: Epigenetic silencing of AATK in acinar to ductal metaplasia in murine model of pancreatic cancer
Source: Clin Epigenetics. 2020 Jun 17;12:87. doi: 10.1186/s13148-020-00878-6 (PMC7301993; doi:10.1186/s13148-020-00878-6)
Supplement: Supplementary file 4 [file 13148_2020_878_MOESM4_ESM.docx]

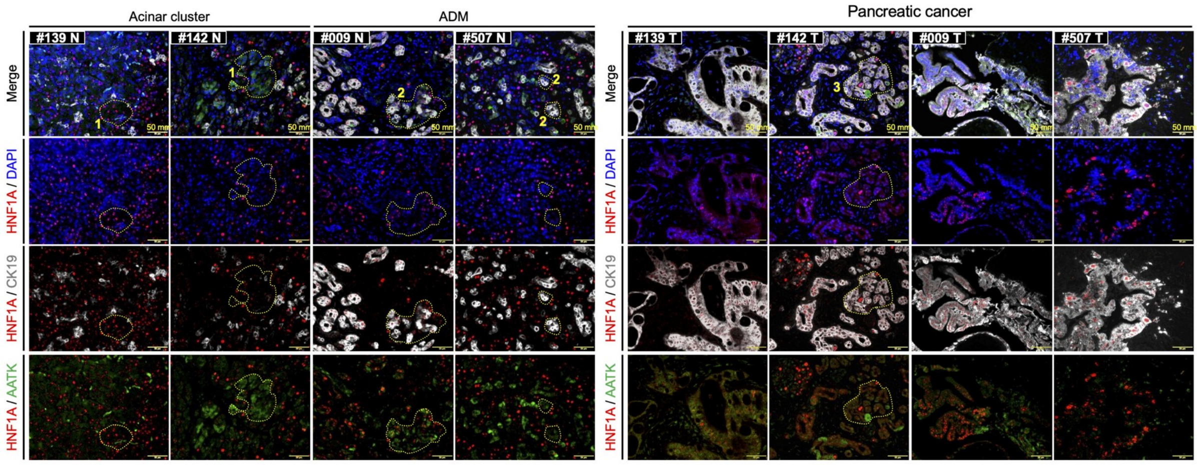

Acinar cluster
ADM
Pancreatic cancer
#139 N
#142 N
#009 N
#507 N
#139 T
#142 T
#009 T
#507 T
Merge
HNF1A / DAPI
HNF1A / CK19
HNF1A / AATK
50 mm
